# 2-(2-Hy­droxy­phen­yl)-3,4-dihydro­iso­quinolin-1(2*H*)-one

**DOI:** 10.1107/S160053681103710X

**Published:** 2011-09-30

**Authors:** Jian Yang, Yanni Ma, Meng Pan, Fangjun Cao, Le Zhou

**Affiliations:** a College of Science, Northwest Agriculture and Forest University, Yangling 712100, People’s Republic of China

## Abstract

There are two independent mol­ecules in the asymmetric unit of the title compound, C_15_H_13_NO_2_, in both the six-membered dihydro­pyridine rings adopt a half-chair conformation. The two benzene rings make dihedral angles of 43.66 (10) and 62.22 (10)° in the two mol­ecules. In the crystal, the two independent mol­ecules are linked alternately by inter­molecular O—H⋯O hydrogen bonds, forming a zigzag chain along the *c* axis. Furthermore, inter­molecular C—H⋯π inter­actions link the chains into a three-dimensional network.

## Related literature

For the synthesis of the title compound, see: Shaw & Zhang (2008 ▶). For the bioactivity of tetra­hydro­isoquinoline derivatives, see: Kamal *et al.* (2011 ▶); Liu *et al.* (2009 ▶); Vrba *et al.* (2009 ▶); Abe *et al.* (2005 ▶); Adhami *et al.* (2004 ▶); Storch *et al.* (2002 ▶).
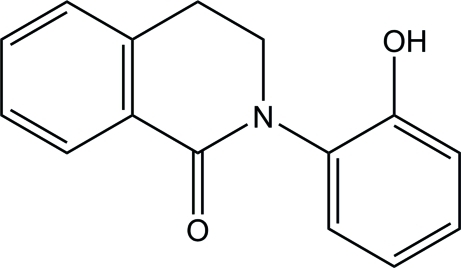


## Experimental

### 

#### Crystal data

C_15_H_13_NO_2_
*M* *_r_* = 239.26
Monoclinic, 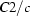

*a* = 21.350 (3) Å
*b* = 11.0670 (14) Å
*c* = 21.064 (3) Å
β = 100.227 (2)°
*V* = 4897.9 (11) Å^3^
*Z* = 16
Mo *K*α radiation
μ = 0.09 mm^−1^
*T* = 296 K
0.50 × 0.35 × 0.34 mm

#### Data collection

Bruker APEXII CCD area-detector diffractometer
Absorption correction: multi-scan (*SADABS*; Sheldrick, 1996 ▶) *T*_min_ = 0.958, *T*_max_ = 0.971
17755 measured reflections
4559 independent reflections
2998 reflections with *I* > 2σ(*I*)
*R*_int_ = 0.034

#### Refinement

*R*[*F* ^2^ > 2σ(*F* ^2^)] = 0.041
*wR*(*F* ^2^) = 0.120
*S* = 1.01
4559 reflections
326 parameters
H-atom parameters constrained
Δρ_max_ = 0.19 e Å^−3^
Δρ_min_ = −0.16 e Å^−3^

### 

Data collection: *APEX2* (Bruker, 2007 ▶); cell refinement: *SAINT* (Bruker, 2007 ▶); data reduction: *SAINT*; program(s) used to solve structure: *SHELXS97* (Sheldrick, 2008 ▶); program(s) used to refine structure: *SHELXL97* (Sheldrick, 2008 ▶); molecular graphics: *SHELXTL* (Sheldrick, 2008 ▶); software used to prepare material for publication: *SHELXTL*.

## Supplementary Material

Crystal structure: contains datablock(s) global, I. DOI: 10.1107/S160053681103710X/is2770sup1.cif

Structure factors: contains datablock(s) I. DOI: 10.1107/S160053681103710X/is2770Isup2.hkl

Supplementary material file. DOI: 10.1107/S160053681103710X/is2770Isup3.cml

Additional supplementary materials:  crystallographic information; 3D view; checkCIF report

## Figures and Tables

**Table 1:** Hydrogen-bond geometry (Å, °) *Cg*2 and *Cg*3 are the centroids of the C1–C6 and C10–C15 rings, respectively.

*D*—H⋯*A*	*D*—H	H⋯*A*	*D*⋯*A*	*D*—H⋯*A*
O2—H2*A*⋯O3^i^	0.82	1.84	2.6388 (18)	166
O4—H4*A*⋯O1	0.82	1.85	2.6668 (17)	175
C7—H7*B*⋯*Cg*2^ii^	0.97	2.92	3.770 (2)	147
C19—H19⋯*Cg*2^iii^	0.93	2.78	3.518 (3)	137
C23—H23*A*⋯*Cg*3^iv^	0.97	2.87	3.771 (2)	155

Symmetry codes: (i) 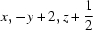; (ii) 
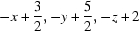
; (iii) 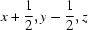; (iv) 
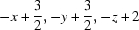
.

**Fig. 1.**
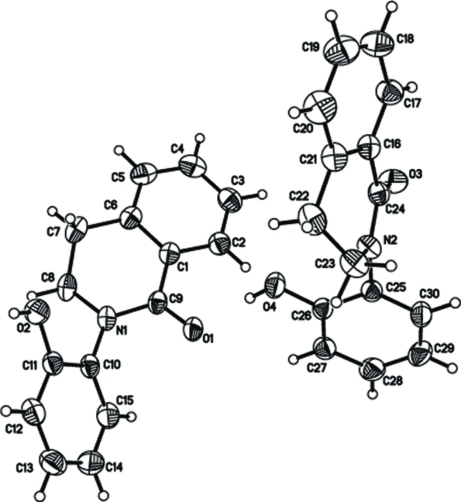
The asymmetric unit of the title compound with 30% probability displacement ellipsoids.

**Fig. 2.**
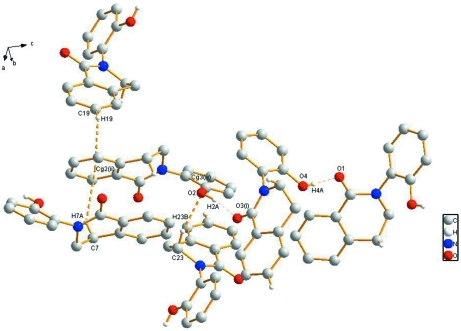
A partial packing diagram of the title compound. Dashed lines indicate the hydrogen bonds and C—H···π interactions.

## supplementary crystallographic information

### Comment

The tetrahydroisoquinoline derivatives have recently attracted a great deal of attention because of their outstanding bioactivity, such as neurotoxicity (Abe *et al.*, 2005; Storch *et al.*, 2002), antitumor activity (Vrba *et al.*, 2009; Adhami *et al.*, 2004), antimicrobial activity (Kamal *et al.*, 2011; Liu *et al.*, 2009), and so on. With the interests in the synthesis of tetrahydroisoquinoline derivatives with bioactivity, we herein report the synthesis and crystal structure of the title compound.

The asymmetric unit of the title compound include two independent molecules (Fig. 1), each is built up from one 3,4-dihydroisoquinolin-1(*2H*)-one fragment connected to one 2-hydroxybenzene ring through the C—N bond. Benzene rings C1—C6 and C10—C15 are inclined with respect to one another with a dihedral angle of 43.66 (10) °, and benzene C16—C21 and C25—C30 with a dihedral angle of 62.22 (10) °. The conformation of the six-membered heterocycle of 3,4-dihydroisoquinolin-1(*2H*)-one fragment is analyzed with respect to the plane formed by C1/C6/C7/C9 and the corresponding deviations of the atoms C8 and N1 are 0.852 and 0.339 Å, respectively. Meanwhile, the corresponding deviations of the atoms C23 and N2 from the plane formed by C16/C21/C22/C24 are 0.761 and 0.289 Å, respectively.

In the crystal structure, the molecules are linked by intermolecular O—H···O hydrogen bonds (Table 1) into a chain along the *c* axis. These chains are further connected by C—H···π interactions (C7—H7B···*Cg*2 and C23—H23A···*Cg*3; Table 1) into a sheet, where *Cg*2 is the centroid of the benzene C1—C6 ring and *Cg*3 is of the benzene C10—C15 ring.  Furthermore, C19—H19···*Cg*2 interaction connects the sheets into three-dimension framework (Fig. 2).

### Experimental

The title compound was synthesized according to the literature procedure (Shaw & Zhang, 2008). An NaOH solution (0.1 mol/*L*, 10 ml) was added to the solution of 2-(2-hydroxyphenyl)-3,4-dihydroisoquinolinum bromide (304 mg, 1 mmol) in ethanol (10 ml) dropwise under stirring. After 24 h, the mixture was extracted by chloroform (30 ml), dried over anhydrous Na_2_SO_4_ and evaporated under reduced pressure. Further purification by silicagel column chromatography (petroleum ether / ethyl acetate = 5:1) and recrystallization gave 85 mg the title compound (yield 36%; m.p. 433–434 K).

### Refinement

H atoms were positioned geometrically and treated as riding, with C—H bond lengths constrained to 0.93 (aromatic CH), or 0.97 Å (methylene CH_2_), and O—H = 0.82 Å, and with *U*_iso_(H) = 1.2*U*_eq_(C) or *U*_iso_(H) = 1.5*U*_eq_(O).

### Crystal data

**Table:**

C_15_H_13_NO_2_	*F*(000) = 2016
*M**_r_* = 239.26	*D*_x_ = 1.298 Mg m^−^^3^
Monoclinic, *C*2/*c*	Mo *K*α radiation, λ = 0.71073 Å
Hall symbol: -C 2yc	Cell parameters from 3281 reflections
*a* = 21.350 (3) Å	θ = 2.5–22.2°
*b* = 11.0670 (14) Å	µ = 0.09 mm^−^^1^
*c* = 21.064 (3) Å	*T* = 296 K
β = 100.227 (2)°	Block, colourless
*V* = 4897.9 (11) Å^3^	0.50 × 0.35 × 0.34 mm
*Z* = 16	

### Data collection

**Table:**

Bruker APEXII CCD area-detector diffractometer	4559 independent reflections
Radiation source: fine-focus sealed tube	2998 reflections with *I* > 2σ(*I*)
graphite	*R*_int_ = 0.034
φ and ω scans	θ_max_ = 25.5°, θ_min_ = 2.4°
Absorption correction: multi-scan (*SADABS*; Sheldrick, 1996)	*h* = −25→25
*T*_min_ = 0.958, *T*_max_ = 0.971	*k* = −13→13
17755 measured reflections	*l* = −25→25

### Refinement

**Table:**

Refinement on *F*^2^	Primary atom site location: structure-invariant direct methods
Least-squares matrix: full	Secondary atom site location: difference Fourier map
*R*[*F*^2^ > 2σ(*F*^2^)] = 0.041	Hydrogen site location: inferred from neighbouring sites
*wR*(*F*^2^) = 0.120	H-atom parameters constrained
*S* = 1.01	*w* = 1/[σ^2^(*F*_o_^2^) + (0.0584*P*)^2^ + 1.0944*P*] where *P* = (*F*_o_^2^ + 2*F*_c_^2^)/3
4559 reflections	(Δ/σ)_max_ = 0.001
326 parameters	Δρ_max_ = 0.19 e Å^−^^3^
0 restraints	Δρ_min_ = −0.16 e Å^−^^3^

### Special details

**Table:**

Geometry. All e.s.d.'s (except the e.s.d. in the dihedral angle between two l.s. planes)are estimated using the full covariance matrix. The cell e.s.d.'s are takeninto account individually in the estimation of e.s.d.'s in distances, anglesand torsion angles; correlations between e.s.d.'s in cell parameters are onlyused when they are defined by crystal symmetry. An approximate (isotropic)treatment of cell e.s.d.'s is used for estimating e.s.d.'s involving l.s. planes.
Refinement. Refinement of *F*^2^ against ALL reflections. The weighted *R*-factor *wR* and goodness of fit *S* are based on *F*^2^, conventional *R*-factors *R* are based on *F*, with *F* set to zero for negative *F*^2^. The threshold expression of *F*^2^ > σ(*F*^2^) is used only for calculating *R*-factors(gt) *etc*. and is not relevant to the choice of reflections for refinement. *R*-factors based on *F*^2^ are statistically about twice as large as those based on *F*, and *R*-factors based on ALL data will be even larger.

### Fractional atomic coordinates and isotropic or equivalent isotropic displacement parameters (Å^2^)

**Table:**

	*x*	*y*	*z*	*U*_iso_*/*U*_eq_	
C1	0.64702 (8)	1.22521 (16)	0.96814 (8)	0.0475 (4)	
C2	0.64055 (9)	1.20334 (19)	0.90234 (9)	0.0557 (5)	
H2	0.6244	1.1297	0.8855	0.067*	
C3	0.65796 (10)	1.2900 (2)	0.86179 (10)	0.0669 (6)	
H3	0.6529	1.2752	0.8177	0.080*	
C4	0.68274 (10)	1.3979 (2)	0.88650 (12)	0.0726 (6)	
H4	0.6942	1.4566	0.8591	0.087*	
C5	0.69074 (10)	1.41978 (19)	0.95193 (12)	0.0665 (6)	
H5	0.7086	1.4924	0.9684	0.080*	
C6	0.67252 (8)	1.33481 (17)	0.99346 (9)	0.0529 (5)	
C7	0.67903 (10)	1.35321 (18)	1.06454 (10)	0.0646 (6)	
H7A	0.6800	1.4390	1.0741	0.077*	
H7B	0.7186	1.3178	1.0863	0.077*	
C8	0.62387 (10)	1.29515 (17)	1.08845 (9)	0.0599 (5)	
H8A	0.6301	1.3014	1.1351	0.072*	
H8B	0.5849	1.3373	1.0707	0.072*	
C9	0.62857 (8)	1.13100 (17)	1.01102 (8)	0.0463 (4)	
C10	0.60218 (8)	1.08107 (17)	1.11477 (8)	0.0490 (4)	
C11	0.64429 (8)	1.06340 (17)	1.17237 (8)	0.0501 (5)	
C12	0.62941 (10)	0.9830 (2)	1.21757 (9)	0.0626 (5)	
H12	0.6573	0.9722	1.2564	0.075*	
C13	0.57360 (11)	0.9192 (2)	1.20525 (11)	0.0732 (6)	
H13	0.5637	0.8650	1.2358	0.088*	
C14	0.53190 (10)	0.9349 (2)	1.14774 (12)	0.0763 (7)	
H14	0.4943	0.8906	1.1394	0.092*	
C15	0.54588 (9)	1.0160 (2)	1.10293 (10)	0.0630 (5)	
H15	0.5174	1.0273	1.0645	0.076*	
C16	0.87970 (8)	0.86788 (16)	0.85716 (8)	0.0486 (4)	
C17	0.91880 (9)	0.92042 (19)	0.81889 (10)	0.0631 (5)	
H17	0.9022	0.9415	0.7765	0.076*	
C18	0.98214 (10)	0.9416 (2)	0.84322 (12)	0.0776 (7)	
H18	1.0083	0.9763	0.8173	0.093*	
C19	1.00639 (11)	0.9113 (2)	0.90587 (13)	0.0839 (7)	
H19	1.0489	0.9268	0.9227	0.101*	
C20	0.96817 (11)	0.8582 (2)	0.94387 (11)	0.0792 (7)	
H20	0.9853	0.8378	0.9862	0.095*	
C21	0.90435 (9)	0.83423 (18)	0.92027 (9)	0.0570 (5)	
C22	0.86093 (10)	0.7762 (2)	0.95990 (9)	0.0669 (6)	
H22A	0.8428	0.8378	0.9839	0.080*	
H22B	0.8852	0.7210	0.9906	0.080*	
C23	0.80834 (10)	0.70835 (19)	0.91819 (9)	0.0610 (5)	
H23A	0.8255	0.6360	0.9017	0.073*	
H23B	0.7771	0.6837	0.9439	0.073*	
C24	0.81068 (9)	0.85581 (16)	0.83172 (8)	0.0477 (4)	
C25	0.70918 (8)	0.77602 (15)	0.84686 (8)	0.0458 (4)	
C26	0.67305 (8)	0.84301 (15)	0.88288 (8)	0.0445 (4)	
C27	0.60732 (9)	0.83775 (18)	0.86810 (9)	0.0555 (5)	
H27	0.5829	0.8822	0.8922	0.067*	
C28	0.57827 (10)	0.7672 (2)	0.81807 (10)	0.0678 (6)	
H28	0.5341	0.7645	0.8082	0.081*	
C29	0.61354 (11)	0.7005 (2)	0.78239 (10)	0.0713 (6)	
H29	0.5933	0.6528	0.7485	0.086*	
C30	0.67912 (10)	0.70412 (18)	0.79681 (9)	0.0613 (5)	
H30	0.7031	0.6583	0.7729	0.074*	
N1	0.61817 (7)	1.16666 (13)	1.06934 (7)	0.0495 (4)	
N2	0.77725 (7)	0.78299 (13)	0.86388 (7)	0.0482 (4)	
O1	0.62296 (6)	1.02339 (11)	0.99405 (6)	0.0565 (3)	
O2	0.69906 (6)	1.12681 (14)	1.18018 (6)	0.0672 (4)	
H2A	0.7209	1.1098	1.2151	0.101*	
O3	0.78604 (6)	0.91442 (13)	0.78357 (6)	0.0650 (4)	
O4	0.70448 (6)	0.91102 (12)	0.93171 (6)	0.0584 (4)	
H4A	0.6787	0.9484	0.9488	0.088*	

### Atomic displacement parameters (Å^2^)

**Table:**

	*U*^11^	*U*^22^	*U*^33^	*U*^12^	*U*^13^	*U*^23^
C1	0.0401 (10)	0.0511 (11)	0.0487 (10)	0.0066 (8)	0.0008 (8)	−0.0025 (9)
C2	0.0509 (11)	0.0617 (12)	0.0527 (11)	0.0019 (9)	0.0040 (9)	−0.0010 (10)
C3	0.0659 (14)	0.0788 (15)	0.0570 (12)	0.0069 (12)	0.0133 (10)	0.0084 (11)
C4	0.0673 (14)	0.0702 (15)	0.0847 (17)	0.0048 (12)	0.0252 (12)	0.0157 (13)
C5	0.0536 (12)	0.0548 (12)	0.0909 (17)	−0.0009 (10)	0.0122 (11)	0.0018 (12)
C6	0.0422 (10)	0.0517 (11)	0.0620 (12)	0.0061 (9)	0.0014 (8)	−0.0054 (10)
C7	0.0681 (13)	0.0524 (12)	0.0678 (13)	−0.0005 (10)	−0.0025 (10)	−0.0135 (10)
C8	0.0706 (13)	0.0551 (12)	0.0510 (11)	0.0115 (10)	0.0029 (9)	−0.0133 (9)
C9	0.0409 (10)	0.0511 (11)	0.0437 (10)	0.0058 (8)	−0.0008 (8)	−0.0085 (9)
C10	0.0434 (10)	0.0593 (11)	0.0445 (10)	0.0055 (9)	0.0084 (8)	−0.0114 (9)
C11	0.0420 (10)	0.0637 (12)	0.0445 (10)	0.0013 (9)	0.0078 (8)	−0.0092 (9)
C12	0.0604 (13)	0.0782 (14)	0.0496 (11)	0.0048 (11)	0.0110 (9)	−0.0009 (10)
C13	0.0717 (15)	0.0833 (16)	0.0704 (15)	−0.0019 (13)	0.0285 (12)	0.0020 (12)
C14	0.0559 (13)	0.0919 (17)	0.0847 (17)	−0.0173 (12)	0.0228 (12)	−0.0132 (14)
C15	0.0455 (11)	0.0835 (15)	0.0593 (12)	0.0020 (11)	0.0071 (9)	−0.0134 (11)
C16	0.0456 (10)	0.0513 (11)	0.0478 (10)	0.0069 (8)	0.0050 (8)	−0.0013 (8)
C17	0.0543 (12)	0.0731 (14)	0.0611 (12)	−0.0016 (10)	0.0079 (10)	0.0040 (10)
C18	0.0510 (13)	0.0902 (17)	0.0914 (17)	−0.0034 (12)	0.0123 (12)	0.0068 (13)
C19	0.0468 (13)	0.0979 (18)	0.1005 (19)	0.0018 (12)	−0.0048 (13)	0.0028 (15)
C20	0.0589 (14)	0.0993 (18)	0.0713 (15)	0.0133 (13)	−0.0106 (12)	0.0074 (13)
C21	0.0527 (12)	0.0615 (12)	0.0536 (11)	0.0133 (10)	0.0008 (9)	0.0018 (9)
C22	0.0708 (14)	0.0796 (15)	0.0481 (11)	0.0173 (12)	0.0045 (10)	0.0127 (10)
C23	0.0627 (13)	0.0640 (12)	0.0561 (12)	0.0143 (10)	0.0099 (10)	0.0180 (10)
C24	0.0498 (11)	0.0527 (11)	0.0400 (9)	0.0053 (9)	0.0066 (8)	−0.0004 (9)
C25	0.0488 (10)	0.0459 (10)	0.0430 (9)	−0.0001 (8)	0.0089 (8)	0.0011 (8)
C26	0.0459 (10)	0.0463 (10)	0.0405 (9)	−0.0012 (8)	0.0052 (8)	−0.0021 (8)
C27	0.0477 (11)	0.0675 (13)	0.0513 (11)	−0.0023 (9)	0.0087 (9)	−0.0026 (9)
C28	0.0552 (12)	0.0857 (15)	0.0598 (13)	−0.0180 (11)	0.0029 (10)	−0.0020 (12)
C29	0.0770 (16)	0.0750 (15)	0.0571 (13)	−0.0239 (12)	−0.0010 (11)	−0.0134 (11)
C30	0.0785 (15)	0.0562 (12)	0.0512 (11)	−0.0020 (11)	0.0167 (10)	−0.0078 (9)
N1	0.0532 (9)	0.0525 (9)	0.0414 (8)	0.0071 (7)	0.0042 (7)	−0.0086 (7)
N2	0.0461 (9)	0.0538 (9)	0.0456 (8)	0.0082 (7)	0.0108 (7)	0.0080 (7)
O1	0.0700 (9)	0.0495 (8)	0.0520 (7)	−0.0015 (6)	0.0165 (6)	−0.0113 (6)
O2	0.0526 (8)	0.0939 (11)	0.0511 (8)	−0.0098 (8)	−0.0021 (6)	0.0017 (7)
O3	0.0563 (8)	0.0822 (10)	0.0511 (8)	−0.0079 (7)	−0.0049 (6)	0.0209 (7)
O4	0.0502 (8)	0.0683 (9)	0.0553 (8)	0.0000 (6)	0.0053 (6)	−0.0208 (7)

### Geometric parameters (Å, °)

**Table:**

C1—C2	1.389 (2)	C16—C21	1.391 (2)
C1—C6	1.396 (3)	C16—C24	1.482 (2)
C1—C9	1.478 (3)	C17—C18	1.378 (3)
C2—C3	1.378 (3)	C17—H17	0.9300
C2—H2	0.9300	C18—C19	1.371 (3)
C3—C4	1.371 (3)	C18—H18	0.9300
C3—H3	0.9300	C19—C20	1.373 (3)
C4—C5	1.380 (3)	C19—H19	0.9300
C4—H4	0.9300	C20—C21	1.390 (3)
C5—C6	1.386 (3)	C20—H20	0.9300
C5—H5	0.9300	C21—C22	1.498 (3)
C6—C7	1.493 (3)	C22—C23	1.498 (3)
C7—C8	1.505 (3)	C22—H22A	0.9700
C7—H7A	0.9700	C22—H22B	0.9700
C7—H7B	0.9700	C23—N2	1.470 (2)
C8—N1	1.477 (2)	C23—H23A	0.9700
C8—H8A	0.9700	C23—H23B	0.9700
C8—H8B	0.9700	C24—O3	1.240 (2)
C9—O1	1.243 (2)	C24—N2	1.338 (2)
C9—N1	1.346 (2)	C25—C30	1.384 (3)
C10—C15	1.385 (3)	C25—C26	1.389 (2)
C10—C11	1.390 (2)	C25—N2	1.436 (2)
C10—N1	1.430 (2)	C26—O4	1.353 (2)
C11—O2	1.349 (2)	C26—C27	1.384 (2)
C11—C12	1.381 (3)	C27—C28	1.368 (3)
C12—C13	1.370 (3)	C27—H27	0.9300
C12—H12	0.9300	C28—C29	1.370 (3)
C13—C14	1.381 (3)	C28—H28	0.9300
C13—H13	0.9300	C29—C30	1.380 (3)
C14—C15	1.373 (3)	C29—H29	0.9300
C14—H14	0.9300	C30—H30	0.9300
C15—H15	0.9300	O2—H2A	0.8200
C16—C17	1.387 (3)	O4—H4A	0.8200
			
C2—C1—C6	119.70 (18)	C18—C17—H17	119.8
C2—C1—C9	119.77 (17)	C16—C17—H17	119.8
C6—C1—C9	120.51 (16)	C19—C18—C17	119.6 (2)
C3—C2—C1	120.46 (19)	C19—C18—H18	120.2
C3—C2—H2	119.8	C17—C18—H18	120.2
C1—C2—H2	119.8	C18—C19—C20	120.3 (2)
C4—C3—C2	119.9 (2)	C18—C19—H19	119.9
C4—C3—H3	120.0	C20—C19—H19	119.9
C2—C3—H3	120.0	C19—C20—C21	121.3 (2)
C3—C4—C5	120.2 (2)	C19—C20—H20	119.3
C3—C4—H4	119.9	C21—C20—H20	119.3
C5—C4—H4	119.9	C20—C21—C16	118.1 (2)
C4—C5—C6	120.8 (2)	C20—C21—C22	123.15 (19)
C4—C5—H5	119.6	C16—C21—C22	118.74 (17)
C6—C5—H5	119.6	C21—C22—C23	111.20 (16)
C5—C6—C1	118.84 (18)	C21—C22—H22A	109.4
C5—C6—C7	123.63 (18)	C23—C22—H22A	109.4
C1—C6—C7	117.53 (17)	C21—C22—H22B	109.4
C6—C7—C8	109.78 (16)	C23—C22—H22B	109.4
C6—C7—H7A	109.7	H22A—C22—H22B	108.0
C8—C7—H7A	109.7	N2—C23—C22	111.26 (17)
C6—C7—H7B	109.7	N2—C23—H23A	109.4
C8—C7—H7B	109.7	C22—C23—H23A	109.4
H7A—C7—H7B	108.2	N2—C23—H23B	109.4
N1—C8—C7	110.58 (15)	C22—C23—H23B	109.4
N1—C8—H8A	109.5	H23A—C23—H23B	108.0
C7—C8—H8A	109.5	O3—C24—N2	122.85 (16)
N1—C8—H8B	109.5	O3—C24—C16	119.94 (17)
C7—C8—H8B	109.5	N2—C24—C16	117.19 (15)
H8A—C8—H8B	108.1	C30—C25—C26	119.71 (17)
O1—C9—N1	121.35 (17)	C30—C25—N2	122.20 (16)
O1—C9—C1	121.52 (16)	C26—C25—N2	118.09 (15)
N1—C9—C1	117.13 (16)	O4—C26—C27	122.76 (16)
C15—C10—C11	119.36 (18)	O4—C26—C25	117.63 (15)
C15—C10—N1	121.76 (17)	C27—C26—C25	119.61 (16)
C11—C10—N1	118.88 (16)	C28—C27—C26	120.02 (19)
O2—C11—C12	123.50 (17)	C28—C27—H27	120.0
O2—C11—C10	116.48 (16)	C26—C27—H27	120.0
C12—C11—C10	120.02 (18)	C27—C28—C29	120.8 (2)
C13—C12—C11	120.0 (2)	C27—C28—H28	119.6
C13—C12—H12	120.0	C29—C28—H28	119.6
C11—C12—H12	120.0	C28—C29—C30	119.91 (19)
C12—C13—C14	120.3 (2)	C28—C29—H29	120.0
C12—C13—H13	119.9	C30—C29—H29	120.0
C14—C13—H13	119.9	C29—C30—C25	119.97 (19)
C15—C14—C13	120.0 (2)	C29—C30—H30	120.0
C15—C14—H14	120.0	C25—C30—H30	120.0
C13—C14—H14	120.0	C9—N1—C10	120.94 (15)
C14—C15—C10	120.2 (2)	C9—N1—C8	120.81 (16)
C14—C15—H15	119.9	C10—N1—C8	118.25 (14)
C10—C15—H15	119.9	C24—N2—C25	120.89 (14)
C17—C16—C21	120.20 (17)	C24—N2—C23	121.64 (15)
C17—C16—C24	119.35 (16)	C25—N2—C23	117.47 (14)
C21—C16—C24	120.32 (17)	C11—O2—H2A	109.5
C18—C17—C16	120.5 (2)	C26—O4—H4A	109.5
			
C6—C1—C2—C3	−1.3 (3)	C24—C16—C21—C22	5.1 (3)
C9—C1—C2—C3	−179.55 (17)	C20—C21—C22—C23	−152.4 (2)
C1—C2—C3—C4	1.0 (3)	C16—C21—C22—C23	28.7 (3)
C2—C3—C4—C5	0.5 (3)	C21—C22—C23—N2	−49.3 (2)
C3—C4—C5—C6	−1.6 (3)	C17—C16—C24—O3	−15.6 (3)
C4—C5—C6—C1	1.3 (3)	C21—C16—C24—O3	160.19 (18)
C4—C5—C6—C7	−179.17 (19)	C17—C16—C24—N2	166.28 (17)
C2—C1—C6—C5	0.2 (3)	C21—C16—C24—N2	−17.9 (3)
C9—C1—C6—C5	178.40 (16)	C30—C25—C26—O4	179.36 (16)
C2—C1—C6—C7	−179.41 (17)	N2—C25—C26—O4	0.2 (2)
C9—C1—C6—C7	−1.2 (2)	C30—C25—C26—C27	−0.4 (3)
C5—C6—C7—C8	144.45 (18)	N2—C25—C26—C27	−179.54 (16)
C1—C6—C7—C8	−36.0 (2)	O4—C26—C27—C28	179.99 (17)
C6—C7—C8—N1	54.5 (2)	C25—C26—C27—C28	−0.3 (3)
C2—C1—C9—O1	18.7 (2)	C26—C27—C28—C29	0.5 (3)
C6—C1—C9—O1	−159.50 (17)	C27—C28—C29—C30	0.0 (3)
C2—C1—C9—N1	−161.35 (16)	C28—C29—C30—C25	−0.6 (3)
C6—C1—C9—N1	20.4 (2)	C26—C25—C30—C29	0.8 (3)
C15—C10—C11—O2	178.12 (17)	N2—C25—C30—C29	179.95 (18)
N1—C10—C11—O2	−2.1 (2)	O1—C9—N1—C10	2.0 (2)
C15—C10—C11—C12	−1.0 (3)	C1—C9—N1—C10	−177.93 (15)
N1—C10—C11—C12	178.74 (16)	O1—C9—N1—C8	−178.89 (16)
O2—C11—C12—C13	−178.00 (19)	C1—C9—N1—C8	1.2 (2)
C10—C11—C12—C13	1.0 (3)	C15—C10—N1—C9	−64.9 (2)
C11—C12—C13—C14	−0.1 (3)	C11—C10—N1—C9	115.33 (18)
C12—C13—C14—C15	−0.8 (3)	C15—C10—N1—C8	115.91 (19)
C13—C14—C15—C10	0.9 (3)	C11—C10—N1—C8	−63.8 (2)
C11—C10—C15—C14	0.0 (3)	C7—C8—N1—C9	−38.9 (2)
N1—C10—C15—C14	−179.70 (18)	C7—C8—N1—C10	140.29 (16)
C21—C16—C17—C18	−1.0 (3)	O3—C24—N2—C25	−4.9 (3)
C24—C16—C17—C18	174.73 (19)	C16—C24—N2—C25	173.20 (15)
C16—C17—C18—C19	−0.5 (3)	O3—C24—N2—C23	175.84 (17)
C17—C18—C19—C20	1.2 (4)	C16—C24—N2—C23	−6.1 (2)
C18—C19—C20—C21	−0.3 (4)	C30—C25—N2—C24	82.4 (2)
C19—C20—C21—C16	−1.3 (3)	C26—C25—N2—C24	−98.5 (2)
C19—C20—C21—C22	179.8 (2)	C30—C25—N2—C23	−98.3 (2)
C17—C16—C21—C20	1.9 (3)	C26—C25—N2—C23	80.9 (2)
C24—C16—C21—C20	−173.83 (18)	C22—C23—N2—C24	40.1 (2)
C17—C16—C21—C22	−179.16 (18)	C22—C23—N2—C25	−139.19 (17)

### Hydrogen-bond geometry (Å, °)

**Table:**

*Cg*2 and *Cg*3 are the centroids of the C1–C6 and C10–C15 rings, respectively.

**Table:**

*D*—H···*A*	*D*—H	H···*A*	*D*···*A*	*D*—H···*A*
O2—H2A···O3^i^	0.82	1.84	2.6388 (18)	166
O4—H4A···O1	0.82	1.85	2.6668 (17)	175
C7—H7B···Cg2^ii^	0.97	2.92	3.770 (2)	147
C19—H19···Cg2^iii^	0.93	2.78	3.518 (3)	137
C23—H23A···Cg3^iv^	0.97	2.87	3.771 (2)	155

Symmetry codes: (i) *x*, −*y*+2, *z*+1/2; (ii) −*x*+3/2, −*y*+5/2, −*z*+2; (iii) *x*+1/2, *y*−1/2, *z*; (iv) −*x*+3/2, −*y*+3/2, −*z*+2.
